# Inherited polymorphisms in the RNA-mediated interference machinery affect microRNA expression and lung cancer survival

**DOI:** 10.1038/sj.bjc.6605976

**Published:** 2010-11-23

**Authors:** M Rotunno, Y Zhao, A W Bergen, J Koshiol, L Burdette, M Rubagotti, R I Linnoila, F M Marincola, P A Bertazzi, A C Pesatori, N E Caporaso, L M McShane, E Wang, M T Landi

**Affiliations:** 1 Division of Cancer Epidemiology and Genetics, National Cancer Institute, National Institutes of Health, 6120 Executive Boulevard, Bethesda, MD 20892-7248, USA; 2 Division of Cancer Treatment and Diagnosis, National Cancer Institute, National Institutes of Health, 6130 Executive Boulevard, Bethesda, MD 20892, USA; 3 Center for Health Sciences, Stanford Research Institute International, 333 Ravenswood Avenue, Menlo Park, CA 94025, USA; 4 EPOCA, Epidemiology Research Center, University of Milan, and Fondazione Istituto Di Ricovero e Cura a Carattere Scientifico, Ospedale Maggiore Policlinico, Mangiagalli e Regina Elena, via Barnaba 8, Milan 20122, Italy; 5 Center for Cancer Research, National Cancer Institute, National Institutes of Health, 37 Convent Drive, MSC 4254, Bethesda, MD 20892, USA; 6 Department of Transfusion Medicine, Clinical Center and Center for Human Immunology, National Institutes of Health, 9000 Rockville Pike, Bethesda, MD 20892, USA

**Keywords:** microRNA biogenesis, polymorphism, lung cancer, survival

## Abstract

**Background:**

MicroRNAs (miRs) have an important role in lung carcinogenesis and progression. Single-nucleotide polymorphisms (SNPs) in genes involved in miR biogenesis may affect miR expression in lung tissue and be associated with lung carcinogenesis and progression.

**Methods:**

We analysed 12 SNPs in *POLR2A*, *RNASEN* and *DICER1* genes in 1984 cases and 2073 controls from the Environment And Genetics in Lung cancer Etiology (EAGLE) study. We investigated miR expression profiles in 165 lung adenocarcinoma (AD) and 125 squamous cell carcinoma tissue samples from the same population. We used logistic and Cox regression models to examine the association of individual genotypes and haplotypes with lung cancer risk and with lung cancer-specific survival, respectively. SNPs-miR expression associations in cases were assessed using two-sample *t*-tests and global permutation tests.

**Results:**

A haplotype in *RNASEN* (*Drosha*) was significantly associated with shorter lung cancer survival (hazard ratio=1.86, 95% CI=1.19–2.92, *P*=0.007). In AD cases, a SNP within the same haplotype was associated with reduced *RNASEN* mRNA expression (*P*=0.013) and with miR expression changes (global *P*=0.007) of miRs known to be associated with cancer (e.g., let-7 family, miR-21, miR-25, miR-126 and miR15a).

**Conclusion:**

Inherited variation in the miR-processing machinery can affect miR expression levels and lung cancer-specific survival.

MicroRNAs (miRs) are small non-coding RNAs that bind to the target transcript in the 3′-UTR and can inhibit the translation of proteins and destabilise their target mRNA (Baek *et al*, 2008). miRs are predicted to regulate ∼30% of the human genome (Lewis *et al*, 2005) including genes in stress resistance, fat metabolism, cell proliferation and apoptosis pathways (Ambros, 2003). Polymorphisms in miR genes or in genes involved in miR biogenesis may affect miR-mediated cell regulation (Mishra and Bertino, 2009; Clague *et al*, 2010). miR biogenesis includes generation of a primary transcript (pri-miR) under RNA polymerase II (*PolR2A*); excision of a stem-loop structure by the nuclear RNaseIII enzyme (*Drosha*) to generate the pre-miR; transportation of the pre-miR to the cytoplasm and processing by another RNaseIII enzyme (*Dicer*) into a ∼22-base mature duplex RNA (Bartel, 2004). An alteration in any step during the maturation process could affect miR production. Impaired miR processing and maturation has been shown to enhance cellular transformation and tumourigenesis (Kumar *et al*, 2008). Given the mounting evidence implicating miRs in lung cancer development and progression (Yanaihara *et al*, 2006; Kumar *et al*, 2008; Landi *et al*, 2010), we investigated the role of single-nucleotide polymorphisms (SNPs) in the RNA-mediated interference machinery involved in miR maturation in lung cancer.

## Materials and methods

We performed SNP genotyping and miR expression profiling using blood and tumour tissue samples from the Environment And Genetics in Lung cancer Etiology (EAGLE) study (Landi *et al*, 2008), including 2100 primary lung cancer cases and 2120 population controls, frequency matched on age, sex and residence, all Caucasians, enrolled in the Lombardy region of Italy in 2002–2005. Institutional review boards of the enrolling hospitals and National Cancer Institute approved the study and participating subjects signed an informed consent.

**Figure 1:**
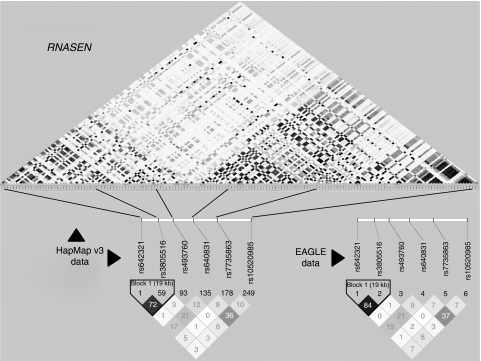
SNPs coverage for the *Drosha* gene. SNPs data available from the HapMap v3 database for the chromosomal region corresponding to the *Drosha* gene. The six SNPs studied in this report are shown in the insets, and linkage disequilibrium (*r*^2^) data from HapMap are compared with data in controls from the EAGLE population showing very similar patterns between the two datasets.

Genomic DNA was isolated from blood samples from 1984 cases and 2073 controls and used to genotype 12 SNPs (Table 1) covering different haplotype blocks in *POLR2A*, *RNASEN* (*Drosha*) and *DICER1* (Figure 1). Genotyping was performed at the Core Genotyping Facility, NCI, using TaqMan assays (http://snp500cancer.nci.nih.gov). Duplicate quality-control samples (2%) showed 100% agreement in all assays. Subjects with at least a 90% genotype call-rate (1946 cases, 1982 controls) were included in the final analyses. All SNPs passed the Hardy–Weinberg equilibrium test among controls (*P*⩾0.26).

The miR expression data were derived from formalin-fixed paraffin-embedded (FFPE) tissue samples in 165 lung adenocarcinoma (AD) and 125 squamous cell carcinoma (SQ) cases from EAGLE, who had not undergone chemotherapy or radiation therapy before tissue collection. Lung cancer histology and the presence of malignant cells in the FFPE tissue blocks were ascertained by the EAGLE local pathologists and were reviewed by a pathologist from the NCI. We excluded tissue blocks with mixed histologies or low frequency of malignant cells. miR expression profiles were obtained using a custom-made two-channel oligo array. The miRs represented on the array, data pre-processing, quality control procedures and selection of the analysed 199 human miRs were described previously (Landi *et al*, 2010). Array results were also confirmed by qRT–PCR using Taqman miRNA assays (Applied Biosystems, Foster City, CA, USA) in 49 samples from EAGLE that had sufficient tumour miR remaining after the array analysis (Landi *et al*, 2010). In addition, we analysed the association between *RNASEN/rs640831* and *RNASEN* gene expression in non-involved lung tissue from 45 AD patients from EAGLE using data from an Affymetrix Chip HG U133A (Affymetrix Inc., Santa Clara, CA, USA).

We tested single SNP and haplotype associations with lung cancer risk in all 3928 subjects and with survival in the 1946 lung cancer cases. In single SNP analyses, homozygosity for the more frequent allele among controls was defined as the reference group and both additive and dominant models were considered. In the haplotype analyses, the most common haplotype was defined as the reference group. The associations between variant genotypes and risk of lung cancer were estimated by odds ratios and their 95% confidence intervals using unconditional logistic regression, adjusted for categories of age, sex, residence, cumulative smoking dose (pack-years), smoking intensity (cigarettes per day), and years-since-quitting smoking. We also performed subgroup analyses by smoking status (never/ever) and major histology types and analyses restricted to patients with resectable tumours (stage I, II and IIIA). Lung cancer-specific survival was defined as time from diagnosis of lung cancer to time of death due to lung cancer. Patients who were still alive at the time of last follow-up (*n*=439) or died for causes unrelated to lung cancer (*n*=98) were censored in the analyses. The association of SNPs and haplotypes with survival-time adjusted for age, sex, stage, and smoking status was estimated by fitting Cox proportional hazards model (Cox, 1972) in patients overall and separately for AD and SQ.

We evaluated the association between miR expression and SNPs within a dominant model in 290 cases using the *t*-test statistic for each miR-SNP combination. For each SNP we counted the number of significant (*P*⩽0.05) miR-SNP associations (n_s_) and then computed a global permutation *P*-value to evaluate the significance of the association between the SNP and the global miR expression profile. The permutation test for each SNP_*i*_ (*i*=1, 2, …, 12) was performed by repeating 9999 times the 199 miR_*j*_−SNP_*i*_^p^ (*j*=1, 2, …, 199) association *t*-tests, where each time SNP_i_^p^ was a random permutation across subjects of the original SNP_*i*_ (*P*=1, 2, …, 9999) data and the number of significant miR_*j*_−SNP_*i*_^p^ associations (n_s_^p^) was recorded for each permutation. The global *P*-value was then defined as one plus the number of times in which n_s_^p^ was at least as large as n_s_ (numerator) divided by the total number of permutations plus one (i.e., denominator=10 000). Finally, we evaluated the association between *RNASEN* mRNA expression and the *rs640831* SNP with a dominant model in 45 AD cases using the *t*-test statistic.

To account for the fact that tests were conducted for 12 different SNPs and 3 haplotypes, we considered 0.01 as *P*-value threshold for statistical significance. All analyses were implemented and performed using the R-project (v2.10) statistical package (http://www.r-project.org/index.html) with the exception of the haplotype analysis, conducted using the THESIAS program (Tregouet and Garelle, 2007).

## Results

None of the investigated polymorphisms in *POLR2A*, *RNASEN* and *DICER1* showed significant association with lung cancer risk or lung cancer survival either overall or by subgroups of histology or smoking status. Analyses based on additive and dominant models gave similar results (Supplementary Materials 1–8). However, we found that a *RNASEN* haplotype, *GTAATC* (frequency=2%), was significantly associated with lung cancer-specific reduced survival compared with the most common haplotype *GTACCT* (frequency=30%) among all cases with hazard ratio (HR)=1.86, 95% CI=1.19–2.92 and *P*=0.007. Similar results were obtained in AD and SQ histological subtypes (HRs=2.33, 3.27; 95% CIs=1.04–5.25, 1.44–7.43; and *P*=0.041, 0.005, respectively) and among ever smoker patients (HR=1.81; 95% CI=1.16–2.82; *P*=0.009). This haplotype-survival association was not observed among the 133 never smoker patients (HR=0.82; 95% CI=0.33–2.06; *P*=0.679). When we repeated this analysis among the 821 patients with early stage resectable tumours (stage=I, II and IIIA), the association between *RNASEN* haplotype *GTAATC* and reduced lung cancer-specific survival was even stronger (HR=2.36, 95% CI=1.36–4.09 and *P*=0.002). Results were also significant in patients with resectable AD and resectable SQ separately (HRs=1.95, 3.99; 95% CIs=1.00–3.81, 1.75–9.11; and *P*=0.05, 0.001, respectively).

We further studied whether the 12 SNPs were associated with expression of mature miRs in lung cancer tissue for all samples and for AD and SQ separately (Table 1). In AD patients, *RNASEN*/*rs640831*, included in the *GTACCT* haplotype, was associated with the expression of 56 miRs (global *P*=0.007, Table 2). On average, for subjects who inherited this SNP, 37 miRs were upregulated and 19 miRs were downregulated in comparison to subjects with the consensus genotype. miRs with tumour suppressor potential (e.g., let-7 family) and miRs with oncogenic or metastatic potential (e.g., miR-21 (Zhu *et al*, 2008), miR-126 (Crawford *et al*, 2008) and miR-15a (Cimmino *et al*, 2005)), were among those with altered expression in the carriers.

We validated the microarray results by qRT–PCR for 4 of the 56 miRs significantly associated with *RNASEN*/*rs640831* in 49 EAGLE lung tumour samples. As shown in Supplementary Figure 1, the correlation was highly significant (*P*=0.001, <0.0001, <0.0001, and 0.002 for let-7g, let-7f, miR-26a and miR-107, respectively). As expected, the correlation between the microarray and qRT–PCR data was inverse as qRT–PCR values are measured in terms of number of measurement cycles needed to reach a certain expression level: the lower the number of cycles the higher the detected expression level. In addition, the association between the expressions as measured by qRT–PCR and *RNASEN*/*rs640831* was qualitatively concordant with the microarray-based results (inverse association in the 24 AD but not in the 23 SQ cases).

Finally, to further elucidate our finding of a correlation between the *RNASEN*/*rs640831* and the miR expression profile among AD cases, we tested the association between *RNASEN* gene expression and the *rs640831* polymorphism in non-involved lung tissue of 45 AD patients from EAGLE. The 25 AD patients carrying one or two *rs640831* minor variants showed a significantly lower mRNA expression than the 20 AD patients homozygous with the more frequent allele (fold change=0.87, *P*=0.013).

## Discussion

We have observed (i) an association between lung cancer survival and a haplotype in *RNASEN,* particularly, among early stage patients and (ii) a differentially expressed miR profile and *RNASEN* gene expression by *RNASEN/rs640831* status in lung tissue. Carrying the minor variant *A vs* the common variant *C* in *RNASEN/rs640831* contributed to the survival association for the *RNASEN* haplotype *GTAATC* compared with the haplotype *GTACCT.* Our results are consistent with the combined effect of multiple genetic markers within a haplotype as better representing the impact of the genetic locus on disease progression than individual markers (Johnson *et al*, 2001; Crawford and Nickerson, 2005). This is the first evidence that inherited variation in the miR-processing machinery, more specifically in *RNASEN*, might affect survival from lung cancer. Previous studies have shown that low *RNASEN* gene expression was associated with survival in oesophageal cancer patients (Sugito *et al*, 2006) and, suggestively, with reduced survival in non-small-cell lung carcinoma patients (Karube *et al*, 2005). Our findings provide a possible genetic basis for the previous reports. The most frequent variant in *RNASEN* was associated with miR expression changes and with lower *RNASEN* mRNA expression in AD. Several of these miRs have been previously reported to be associated with lung cancer survival in the EAGLE study (Landi *et al*, 2010) and other lung cancer studies (Markou *et al*, 2008; Yu *et al*, 2008; Raponi *et al*, 2009). An analogous global modification of miR profile due to changes in *Drosha* transcript expression level has been observed in cervical cancer (Muralidhar *et al*, 2007). A similar finding was not observed in our analysis restricted to cases with SQ histology, suggesting that the mechanism by which *RNASEN/rs640831* affects miR expression is specific to AD lung tissues. Such observation is consistent with previous studies showing that protein levels of genes involved in the endogenous miR machinery differ between lung AD and SQ histology subtypes (Chiosea *et al*, 2007). It is also possible that the relatively small sample size of the SQ affected the results.

In contrast to the survival analysis that was based on a large sample (*n*∼2000), allowing for exploration of several models, including haplotypes, the miR expression analysis was based on a smaller sample size (*n*∼300, of which 125 were SQ). Consequently, only the most informative SNP (MAF=34%) could be adequately tested with the miR profile. In contrast, we were not able to detect possible effects of the full haplotype in association with the miR profile, because this test would have required hundreds of samples with miR expression data. Further functional studies and/or larger miR profiling studies are necessary to confirm our findings.

In conclusion, in the largest study of SNPs in genes involved in miR biogenesis and lung cancer to date, an inherited variant in the *RNASEN* gene, coding for the *Drosha* enzyme, was associated with reduced expression of the *Drosha* gene and changes in the expression of miRs involved in many cancer-related processes. A haplotype in *Drosha* that included the inherited *RNASEN* variant and five additional variants was associated with poor lung cancer survival, particularly, among early stage patients. *Drosha* gene variants may affect miR-processing machinery including miR maturation and expression level, which may consequently affect the function of miRs in transcript and protein translation regulation. These variants in *Drosha* may have potential prognostic implications in lung cancer.

## Figures and Tables

**Table 1:** SNPs in the RNA-mediated interference machinery and correlation with miR expression

					**All samples**				**AD samples**				**SQ samples**			
**Gene**	**SNP**	**Position**	**Alleles**	**MAF**	**Ref.**	**MA**	**miR**	***P*-value**	**Ref.**	**MA**	**miR**	***P*-value**	**Ref.**	**MA**	**miR**	***P*-value**
*RNASEN*	*rs642321*	Chr5:31436760	G:A	0.21	168	108	20	0.125	101	59	15	0.205	67	49	10	0.365
*RNASEN*	*rs3805516*	Chr5:31456427	T:C	0.22	163	111	25	0.082	97	62	11	0.295	66	49	17	0.155
*RNASEN*	*rs493760*	Chr5:31472797	A:G	0.29	144	132	3	0.851	77	82	3	0.841	67	50	4	0.795
*RNASEN*	*rs640831*	Chr5:31495192	C:A	0.34	123	152	30	0.056	68	92	**56**	**0.007**	55	60	1	0.985
*RNASEN*	*rs7735863*	Chr5:31522297	C:T	0.10	223	49	9	0.393	130	26	5	0.656	93	23	9	0.429
*RNASEN*	*rs10520985*	Chr5:31560322	C:T	0.46	78	193	10	0.351	47	109	10	0.335	31	84	2	0.946
*DICER1*	*rs1209904*	Chr14:94633465	G:A	0.26	139	137	4	0.757	80	79	20	0.123	59	58	2	0.944
*DICER1*	*rs2297730*	Chr14:94648428	A:G	0.08	230	45	12	0.276	139	19	9	0.393	91	26	6	0.626
*POLR2A*	*rs8065577*	Chr17:7325898	G:C	0.24	165	112	2	0.929	100	60	3	0.840	65	52	3	0.877
*POLR2A*	*rs7217258*	Chr17:7336273	A:G	0.28	145	132	5	0.658	87	73	4	0.746	58	59	2	0.944
*POLR2A*	*rs2071504*	Chr17:7346661	C:T	0.10	215	58	3	0.852	123	34	2	0.928	92	24	9	0.420
*POLR2A*	*rs6761*	Chr17:7358387	T:C	0.33	123	151	1	0.982	75	83	2	0.927	48	68	2	0.945

Abbreviations: miR=microRNAs; SNP=single-nucleotide polymorphism.

Gene name, rsID, chromosomal location, major : minor alleles and minor allele frequency (MAF) of the studied SNPs are reported in the first five columns. The remaining columns show the results for the analysis of correlation between each SNP and the expression of 199 human miRs using all samples and restricted to adenocarcinoma (AD) and squamous cell carcinoma (SQ) lung cancer tissue samples. For each SNP and analysis type, we reported the number of subjects homozygous for the most common allele (used as reference and indicated with ‘Ref.’), the number carrying one or two minor variant alleles (indicated with ‘MA’), the number of miRs individually correlated with the SNP at a significant level 0.05 (indicated with ‘miR’), and the global *P*-value based on 10 000 permutations for the association of the SNP with the miR profile. Bold indicates a significant correlation (global *P*-value <0.01).

**Table 2:** miRs significantly correlated with *RNASEN* SNP *rs640831* in AD patients

**MicroRNAs**	**Unique ID**	***P*-value**	**Fold change**	**Mean in Ref. (log_2_)**	**Mean in MA (log_2_)**
*Upregulated*					
has-miR-30b	MIMAT0000420	0.0003	1.72	−2.60	−1.82
**hsa-miR-25**	MIMAT0000081	0.0011	1.57	−2.07	−1.41
hsa-miR-92	MIMAT0000092	0.0013	1.51	−4.07	−3.48
**hsa-let-7g**	MIMAT0000414	0.0016	1.68	−2.81	−2.06
**hsa-miR-21**	MIMAT0000076	0.0016	1.89	−3.39	−2.47
hsa-miR-200c	MIMAT0000617	0.0020	1.47	0.90	1.46
hsa-miR-106a	MIMAT0000103	0.0020	1.45	−4.02	−3.48
hsa-miR-30c	MIMAT0000244	0.0022	1.68	−2.65	−1.90
hsa-miR-30a-5p	MIMAT0000087	0.0025	1.48	−1.18	−0.62
**hsa-let-7b**	MIMAT0000063	0.0025	1.50	−1.22	−0.64
**hsa-let-7f**	MIMAT0000067	0.0031	1.65	−2.74	−2.02
hsa-miR-181a	MIMAT0000256	0.0031	1.38	−0.90	−0.43
hsa-miR-20b	MIMAT0001413	0.0036	1.38	−3.52	−3.06
hsa-miR-103	MIMAT0000101	0.0036	1.37	−1.60	−1.14
hsa-miR-98	MIMAT0000096	0.0038	1.62	−2.33	−1.63
**hsa-let-7c**	MIMAT0000064	0.0040	1.53	−1.76	−1.15
hsa-miR-20a	MIMAT0000075	0.0045	1.45	−4.64	−4.11
**hsa-miR-26a**	MIMAT0000082	0.0070	1.65	−1.01	−0.29
hsa-miR-29a	MIMAT0000086	0.0071	1.52	−1.82	−1.22
**hsa-miR-126**	MIMAT0000445	0.0074	1.55	2.23	2.86
hsa-miR-17-5p	MIMAT0000070	0.0096	1.33	−3.71	−3.30
hsa-miR-107	MIMAT0000104	0.0104	1.30	−1.54	−1.16
hsa-miR-19b	MIMAT0000074	0.0127	1.41	−4.20	−3.70
**hsa-let-7a**	MIMAT0000062	0.0182	1.40	−2.05	−1.57
hsa-miR-106b	MIMAT0000680	0.0198	1.27	−3.32	−2.98
**hsa-let-7i**	MIMAT0000415	0.0199	1.39	−2.15	−1.68
hsa-miR-200b	MIMAT0000318	0.0205	1.46	1.67	2.21
hsa-miR-143	MIMAT0000435	0.0205	1.18	0.88	1.12
hsa-miR-26b	MIMAT0000083	0.0210	1.39	−1.46	−0.98
**hsa-miR-15a**	MIMAT0000068	0.0244	1.33	−2.04	−1.62
hsa-miR-30d	MIMAT0000245	0.0281	1.28	−0.79	−0.44
hsa-miR-93	MIMAT0000093	0.0285	1.30	−3.36	−2.98
hsa-miR-23a	MIMAT0000078	0.0348	1.26	0.36	0.69
hsa-miR-125a	MIMAT0000443	0.0377	1.21	0.68	0.96
hsa-miR-22	MIMAT0000077	0.0415	1.30	0.55	0.93
**hsa-miR-146b**	MIMAT0002809	0.0422	1.36	−4.46	−4.02
hsa-miR-429	MIMAT0001536	0.0432	1.36	−0.43	0.02
					
*Downregulated*					
hsa-miR-452	MIMAT0001635	0.0003	0.88	0.18	0.00
**hsa-miR-370**	MIMAT0000722	0.0024	0.90	0.41	0.26
hsa-miR-122a	MIMAT0000421	0.0037	0.89	0.14	−0.03
hsa-miR-130b	MIMAT0000691	0.0044	0.92	−0.11	−0.23
hsa-miR-510	MIMAT0002882	0.0047	0.91	0.22	0.08
hsa-miR-188	MIMAT0000457	0.0065	0.91	0.18	0.04
hsa-miR-509	MIMAT0002881	0.0117	0.86	−0.40	−0.62
hsa-miR-198	MIMAT0000228	0.0134	0.90	0.43	0.28
hsa-miR-485-5p	MIMAT0002175	0.0134	0.90	0.81	0.65
hsa-miR-518c^*^	MIMAT0002847	0.0145	0.89	−0.01	−0.17
hsa-mir-610	MIMAT0003278	0.0162	0.83	1.01	0.73
hsa-miR-488	MIMAT0002804	0.0166	0.91	−0.76	−0.90
**hsa-miR-453**	MIMAT0001630	0.0353	0.93	0.38	0.28
hsa-mir-628	MIMAT0003297	0.0405	0.89	−0.14	−0.30
**hsa-miR-432**	MIMAT0002814	0.0420	0.89	−0.02	-0.20
hsa-mir-623	MIMAT0003292	0.0429	0.88	−0.05	−0.24
**hsa-miR-299-3p**	MIMAT0000687	0.0473	0.92	0.05	−0.07
hsa-miR-524^*^	MIMAT0002849	0.0473	0.93	0.07	−0.03
hsa-miR-383	MIMAT0000738	0.0498	0.91	0.83	0.70

Abbreviations: AD=adenocarcinoma; miR=microRNA; SNP=single-nucleotide polymorphism.

The 56 miRs significantly correlated with *RNASEN/rs640831* in AD patients are listed ranking by *P*-value of each SNP-miR correlation. The analysed miR data is a miR expression intensity ratio between the examined miR and the reference EBV cell line, followed by median normalisation and log_2_ base transformation (i.e., a negative value indicates a ratio between 0 and 1). For each miR we also reported the fold change of the expression ratio for minor allele carriers (indicated with ‘MA’) compared with major allele homozygotes (indicated with ‘Ref.’), and the expression ratio means in the two compared groups. miRs whose expression has been associated with lung cancer in previous studies are shown in bold. The asterisk (^*^) symbol after a miR label designates a complementary miR.

## References

[bib1] Ambros V (2003) MicroRNA pathways in flies and worms: growth, death, fat, stress, and timing. Cell 113: 673–676 12809598 10.1016/s0092-8674(03)00428-8

[bib2] Baek D, Villen J, Shin C, Camargo FD, Gygi SP, Bartel DP (2008) The impact of microRNAs on protein output. Nature 455: 64–71 18668037 10.1038/nature07242 PMC2745094

[bib3] Bartel DP (2004) MicroRNAs: genomics, biogenesis, mechanism, and function. Cell 116: 281–297 14744438 10.1016/s0092-8674(04)00045-5

[bib4] Chiosea S, Jelezcova E, Chandran U, Luo J, Mantha G, Sobol RW, Dacic S (2007) Overexpression of Dicer in precursor lesions of lung adenocarcinoma. Cancer Res 67: 2345–2350 17332367 10.1158/0008-5472.CAN-06-3533

[bib5] Cimmino A, Calin GA, Fabbri M, Iorio MV, Ferracin M, Shimizu M, Wojcik SE, Aqeilan RI, Zupo S, Dono M, Rassenti L, Alder H, Volinia S, Liu CG, Kipps TJ, Negrini M, Croce CM (2005) miR-15 and miR-16 induce apoptosis by targeting BCL2. Proc Natl Acad Sci USA 102: 13944–13949 16166262 10.1073/pnas.0506654102 PMC1236577

[bib6] Clague J, Lippman SM, Yang H, Hildebrandt MA, Ye Y, Lee JJ, Wu X (2010) Genetic variation in MicroRNA genes and risk of oral premalignant lesions. Mol Carcinog 49: 183–189 19851984 10.1002/mc.20588 PMC3640857

[bib7] Cox D (1972) Regression models and life tables (with discussion). J Roy Stat Soc B 4: 187–220

[bib8] Crawford DC, Nickerson DA (2005) Definition and clinical importance of haplotypes. Annu Rev Med 56: 303–320 15660514 10.1146/annurev.med.56.082103.104540

[bib9] Crawford M, Brawner E, Batte K, Yu L, Hunter MG, Otterson GA, Nuovo G, Marsh CB, Nana-Sinkam SP (2008) MicroRNA-126 inhibits invasion in non-small cell lung carcinoma cell lines. Biochem Biophys Res Commun 373: 607–612 18602365 10.1016/j.bbrc.2008.06.090

[bib10] Johnson GC, Esposito L, Barratt BJ, Smith AN, Heward J, Di GG, Ueda H, Cordell HJ, Eaves IA, Dudbridge F, Twells RC, Payne F, Hughes W, Nutland S, Stevens H, Carr P, Tuomilehto-Wolf E, Tuomilehto J, Gough SC, Clayton DG, Todd JA (2001) Haplotype tagging for the identification of common disease genes. Nat Genet 29: 233–237 11586306 10.1038/ng1001-233

[bib11] Karube Y, Tanaka H, Osada H, Tomida S, Tatematsu Y, Yanagisawa K, Yatabe Y, Takamizawa J, Miyoshi S, Mitsudomi T, Takahashi T (2005) Reduced expression of Dicer associated with poor prognosis in lung cancer patients. Cancer Sci 96: 111–115 15723655 10.1111/j.1349-7006.2005.00015.x PMC11158408

[bib12] Kumar MS, Erkeland SJ, Pester RE, Chen CY, Ebert MS, Sharp PA, Jacks T (2008) Suppression of non-small cell lung tumor development by the let-7 microRNA family. Proc Natl Acad Sci USA 105: 3903–3908 18308936 10.1073/pnas.0712321105 PMC2268826

[bib13] Landi MT, Consonni D, Rotunno M, Bergen AW, Goldstein AM, Lubin JH, Goldin L, Alavanja M, Morgan G, Subar AF, Linnoila I, Previdi F, Corno M, Rubagotti M, Marinelli B, Albetti B, Colombi A, Tucker M, Wacholder S, Pesatori AC, Caporaso NE, Bertazzi PA (2008) Environment and genetics in lung cancer etiology (EAGLE) study: an integrative population-based case-control study of lung cancer. BMC Public Health 8: 203 18538025 10.1186/1471-2458-8-203 PMC2464602

[bib14] Landi MT, Zhao Y, Rotunno M, Koshiol J, Liu H, Bergen AW, Rubagotti M, Goldstein AM, Linnoila I, Marincola FM, Tucker MA, Bertazzi PA, Pesatori AC, Caporaso NE, McShane LM, Wang E (2010) MicroRNA expression differentiates histology and predicts survival of lung cancer. Clin Cancer Res 16: 430–441 20068076 10.1158/1078-0432.CCR-09-1736 PMC3163170

[bib15] Lewis BP, Burge CB, Bartel DP (2005) Conserved seed pairing, often flanked by adenosines, indicates that thousands of human genes are microRNA targets. Cell 120: 15–20 15652477 10.1016/j.cell.2004.12.035

[bib16] Markou A, Tsaroucha EG, Kaklamanis L, Fotinou M, Georgoulias V, Lianidou ES (2008) Prognostic value of mature microRNA-21 and microRNA-205 overexpression in non-small cell lung cancer by quantitative real-time RT-PCR. Clin Chem 54: 1696–1704 18719201 10.1373/clinchem.2007.101741

[bib17] Mishra PJ, Bertino JR (2009) MicroRNA polymorphisms: the future of pharmacogenomics, molecular epidemiology and individualized medicine. Pharmacogenomics 10: 399–416 19290790 10.2217/14622416.10.3.399 PMC2705205

[bib18] Muralidhar B, Goldstein LD, Ng G, Winder DM, Palmer RD, Gooding EL, Barbosa-Morais NL, Mukherjee G, Thorne NP, Roberts I, Pett MR, Coleman N (2007) Global microRNA profiles in cervical squamous cell carcinoma depend on Drosha expression levels. J Pathol 212: 368–377 17471471 10.1002/path.2179

[bib19] Raponi M, Dossey L, Jatkoe T, Wu X, Chen G, Fan H, Beer DG (2009) MicroRNA classifiers for predicting prognosis of squamous cell lung cancer. Cancer Res 69: 5776–5783 19584273 10.1158/0008-5472.CAN-09-0587

[bib20] Sugito N, Ishiguro H, Kuwabara Y, Kimura M, Mitsui A, Kurehara H, Ando T, Mori R, Takashima N, Ogawa R, Fujii Y (2006) RNASEN regulates cell proliferation and affects survival in esophageal cancer patients. Clin Cancer Res 12: 7322–7328 17121874 10.1158/1078-0432.CCR-06-0515

[bib21] Tregouet DA, Garelle V (2007) A new JAVA interface implementation of THESIAS: testing haplotype effects in association studies. Bioinformatics 23: 1038–1039 17308338 10.1093/bioinformatics/btm058

[bib22] Yanaihara N, Caplen N, Bowman E, Seike M, Kumamoto K, Yi M, Stephens RM, Okamoto A, Yokota J, Tanaka T, Calin GA, Liu CG, Croce CM, Harris CC (2006) Unique microRNA molecular profiles in lung cancer diagnosis and prognosis. Cancer Cell 9: 189–198 16530703 10.1016/j.ccr.2006.01.025

[bib23] Yu SL, Chen HY, Chang GC, Chen CY, Chen HW, Singh S, Cheng CL, Yu CJ, Lee YC, Chen HS, Su TJ, Chiang CC, Li HN, Hong QS, Su HY, Chen CC, Chen WJ, Liu CC, Chan WK, Chen WJ, Li KC, Chen JJ, Yang PC (2008) MicroRNA signature predicts survival and relapse in lung cancer. Cancer Cell 13: 48–57 18167339 10.1016/j.ccr.2007.12.008

[bib24] Zhu S, Wu H, Wu F, Nie D, Sheng S, Mo YY (2008) MicroRNA-21 targets tumor suppressor genes in invasion and metastasis. Cell Res 18: 350–359 18270520 10.1038/cr.2008.24

