# Supplementary material for: Inherited polymorphisms in the RNA-mediated interference machinery affect microRNA expression and lung cancer survival
Source: Br J Cancer. 2010 Nov 23;103(12):1870–4. doi: 10.1038/sj.bjc.6605976 (PMC3008605; doi:10.1038/sj.bjc.6605976)
Supplement: Supplementary Figure 1 [file 6605976x1.doc]

**Supplemental Figure 1. Validation of microarray results by qRT-PCR Taqman assays.**

We validated the microarray results by qRT-PCR Taqman assays for 4 of the miRs significantly associated with *RNASEN*/*rs640831* in 49 EAGLE samples from 24 adenocarcinoma (AD) and 23 squamous cell carcinoma (SQ) patients. Each row in the figure shows results for each of the 4 miRs: let-7g (first row), let-7f (second row), miR-26a (third row), and miR-107 (fourth row). The 4 panels on the left show the correlation between microarray expression (x axis) and qRT-PCR expression (y axis) of the 4 miRs. The correlation is significantly negative (based on a Pearson correlation test), as expected given that qRT-PCR is measured in terms of number of measurement cycles needed to reach a certain expression level: the lower the number of cycles the higher the detected expression level. The other panels are boxplots (i.e., the smallest observation, lower quartile, median, upper quartile, and largest observation) for the miRs expression as measured by qRT-PCR (y axis) by *RNASEN*/*rs640831* status (x axis, where 0 indicates subjects with 2 major allele and 1 indicates subjects with 1 or 2 minor alleles), among AD patients (central panels) and SQ patients (right panels). The association based on a t-test between the miRs expression and this *RNASEN*/*rs640831* was qualitatively concordant with the microarray-based results (inverse association with the SNP in AD but not in SQ cases), although not significantly, possibly because of the low sample size. FC indicates the fold change and p indicates the Pearson correlation and t-test p-values.
